# Measurement of Phospholipids May Improve Diagnostic Accuracy in Ovarian Cancer

**DOI:** 10.1371/journal.pone.0046846

**Published:** 2012-10-17

**Authors:** Lian Shan, Y. Ann Chen, Lorelei Davis, Gang Han, Weiwei Zhu, Ashley D. Molina, Hector Arango, James P. LaPolla, Mitchell S. Hoffman, Thomas Sellers, Tyler Kirby, Santo V. Nicosia, Rebecca Sutphen

**Affiliations:** 1 Frantz Biomarkers, LLC, Mentor, Ohio, United States of America; 2 Biostatistics, Moffitt Cancer Center and Research Institute, Tampa, Florida, United States of America; 3 Epidemiology Center, Morsani College of Medicine, University of South Florida, Tampa, Florida, United States of America; 4 West Coast Gynecologic Oncology, Clearwater, Florida, United States of America; 5 Women's Cancer Associates, Gynecologic Oncology, St. Petersburg, Florida, United States of America; 6 Gynecologic Oncology, College of Medicine, University of South Florida, Tampa, Florida, United States of America; 7 Cancer Epidemiology, Moffitt Cancer Center and Research Institute, Tampa, Florida, United States of America; 8 Department of Pathology and Cell Biology, College of Medicine, University of South Florida, Tampa, Florida, United States of America; The Chinese University of Hong Kong, Hong Kong

## Abstract

**Background:**

More than two-thirds of women who undergo surgery for suspected ovarian neoplasm do not have cancer. Our previous results suggest phospholipids as potential biomarkers of ovarian cancer. In this study, we measured the serum levels of multiple phospholipids among women undergoing surgery for suspected ovarian cancer to identify biomarkers that better predict whether an ovarian mass is malignant.

**Methodology/Principal Findings:**

We obtained serum samples preoperatively from women with suspected ovarian cancer enrolled through a prospective, population-based rapid ascertainment system. Samples were analyzed from all women in whom a diagnosis of epithelial ovarian cancer (EOC) was confirmed and from benign disease cases randomly selected from the remaining (non-EOC) samples. We measured biologically relevant phospholipids using liquid chromatography/electrospray ionization mass spectrometry. We applied a powerful statistical and machine learning approach, Hybrid huberized support vector machine (HH-SVM) to prioritize phospholipids to enter the biomarker models, and used cross-validation to obtain conservative estimates of classification error rates.

**Results:**

The HH-SVM model using the measurements of specific combinations of phospholipids supplements clinical CA125 measurement and improves diagnostic accuracy. Specifically, the measurement of phospholipids improved sensitivity (identification of cases with preoperative CA125 levels below 35) among two types of cases in which CA125 performance is historically poor - early stage cases and those of mucinous histology. Measurement of phospholipids improved the identification of early stage cases from 65% (based on CA125) to 82%, and mucinous cases from 44% to 88%.

**Conclusions/Significance:**

Levels of specific serum phospholipids differ between women with ovarian cancer and those with benign conditions. If validated by independent studies in the future, these biomarkers may serve as an adjunct at the time of clinical presentation, to distinguish between women with ovarian cancer and those with benign conditions with shared symptoms and features.

## Introduction

Data suggest that among women with newly diagnosed ovarian cancer, those whose initial surgery is performed by a gynecologic oncologist have lower morbidity and mortality and increased overall survival [1], [2]. However, to date in the U.S., initial surgery for suspected ovarian cancer is often performed without referral to such specialists [1]. This is partly because there is no accurate way to know in advance of surgery whether a pelvic mass suspected to be ovarian cancer is, in fact, cancer. As a result, more than two-thirds of women who undergo surgery for suspected ovarian neoplasm do not have cancer [3]–[5]. Since it is estimated that 5–10% of U.S. women will undergo a surgical procedure for suspected ovarian neoplasm during their lifetime, this is an issue with significant public health impact [6].

It is currently difficult to appropriately triage women with a pelvic mass to gynecologic oncologists based on a high index of suspicion for ovarian cancer, since there are limited tools to perform such an assessment. A new blood test, OVA1, recently received FDA approval as an adjunct in presurgical evaluation of adnexal masses [7]. The only well-validated ovarian cancer biomarker in clinical use, serum CA125, is elevated in only approximately half of early stage epithelial ovarian cancer (EOC) and is not elevated in approximately 20% of all stage EOC, rendering it insufficiently sensitive [8], [9].The most commonly reported CA125 reference value that designates a clinically positive screening test is 35 units/ml although CA125 is also elevated in many benign gynecologic diseases, including many conditions associated with pelvic masses. One of the first few screening studies combining CA125 and ultrasound has shown that using serum CA125 as the first line test and pelvic ultrasound as a secondary test has high specificity (99.9%) and positive predictive value (26.8%) for detecting ovarian cancer [10]. In a retrospective analysis of high-risk women, the use of repeated measurements of CA125 values incorporated in longitudinal statistical models showed an improved sensitivity from 70% to 86% while maintaining specificity at 98% [11]. A recent large prospective trial (UK Collaborative Trial of Ovarian Screening [UKCTOC]), assessing multimodality screening (annual CA125 screening interpreted using an algorithm for risk of ovarian cancer along with transvaginal ultrasound scan as a second line test) suggests that multimodality has significantly higher specificity (99.8%) than using ultrasound alone (98.2%) for detecting primary ovarian and tubal cancers, although no statistically significant difference in sensitivity was found [12]. In the Prostate, Lung, Colorectal and Ovarian Cancer Screening trial, a randomized trial, the results through four rounds of ovarian cancer screening showed that most of the screen-detected cases were late-stage [13], which supports the need for additional methods and strategies to achieve early detection. Thus, a tool or biomarker allowing accurate classification of patients into high and low risk groups for ovarian malignancy would improve the ability to appropriately triage patients to gynecologic oncologists. Additionally, in individual cases of pelvic mass where clinical suspicion of malignancy is not high, it is possible that biomarker evaluation might facilitate watchful waiting and result in fewer and/or less urgent surgeries.

We previously analyzed circulating phospholipids, including lysophosphatidic acid (LPA), lysophosphatidylcholine (LPC) and related species, for their potential to discriminate between women with EOC and healthy controls, with promising results [14]. In other recent work, we identified plasmalogens as another group of circulating substances with potential as ovarian cancer biomarkers [15]. In the present study, we sought to expand on our earlier work toward validation of these promising biomarkers for clinical application in the diagnosis of ovarian cancer. We measured the serum levels of multiple lipid species, including particular species of LPA, LPC and plasmenylphosphoethanolamine (PPE) in order to assess their performance in discriminating between EOC and benign disease compared with, or in combination with, clinical measurement of CA125 in preoperative samples obtained prospectively from women presenting with suspected ovarian cancer.

Some of the general computational challenges for biomarker studies include the following: identifying powerful statistical methods, selecting predictive markers from a (large) panel of potential candidates, evaluating the joint effects of multiple markers, and avoiding model overfitting due to the complexity of nonlinear computational models. Support vector machine (SVM) has been shown to have superior performance in terms of classification accuracy and has been identified as one of the most powerful statistical and machine learning methods to analyze high-dimensional data, such as that derived from gene expression and biomarker studies [16], [17]. In our study, we have employed SVM. To prioritize the markers entering the model, we first used Hybrid huberized support vector machines (HH-SVM), which automatically select variables and estimate their importance with efficient computational cost [18]. To avoid model overfitting, we use a common resampling technique, five-fold cross-validation, to obtain more objective error rates [19]. Briefly, we first fitted the model using 4/5 of the data while testing the results on the remaining 1/5 of the data, and this step was repeated 5 times so that each 1/5 of the data was validated once during the model development. We have developed two types of models in this study, one-step models and two-step models. To develop our one-step models, CA125 was included along with the other measured biomarkers as continuous variables with no pre-specified cutpoint. To develop the two-step models, we first employed the commonly reported clinical reference value of 35 units/ml for CA125 that designates a positive screening test and then employed our algorithm for the other measured biomarkers in order to query whether an additional test added to this reference value of CA125 could improve diagnostic accuracy for classifying, among women with a clinical presentation of suspected ovarian cancer, between “cases” (samples from women in whom surgery confirmed EOC) and “benigns” (samples from women in whom surgery confirmed no ovarian cancer). More details for the model development are provided in the statistical section.

## Materials and Methods

### Subjects

The study protocol was reviewed and approved by the Institutional Review Board at the University of South Florida and all participants provided written informed consent. Subjects in the current study were enrolled through an ongoing prospective population-based investigation of ovarian cancer in the Tampa, Florida metropolitan area (population approximately 2 million). Through the study's rapid ascertainment system, a total of 1057 women with suspected ovarian cancer were enrolled preoperatively between January 2005 and March 2009, accounting for an estimated 75% of all eligible cases in the defined geographic region. Women with a prior unilateral or bilateral oophorectomy were ineligible, as were women with a previous history of cancer (except for non-melanoma skin cancer). All patients underwent preoperative radiologic imaging, either by pelvic ultrasound, CT, and/or MRI. Only patients who underwent surgery based on clinical suspicion of ovarian cancer were eligible and if a patient was diagnosed with EOC, surgical staging was documented (including 233 in whom EOC was confirmed - defined as primary ovarian, primary fallopian tube or primary peritoneal cancer). The benign samples were randomly selected from the remaining (non-EOC) samples; benign pathologies include endometriosis, ovarian cyst, and ovarian fibroma. Preoperative serum CA125 clinical measurements were obtained from medical records. Pathology was centrally reviewed by an expert ovarian cancer pathologist (S.N). All histologic evaluations were performed blinded to the laboratory values of the biomarker assays and all laboratory testing was performed blinded to histologic outcome (benign versus EOC). Samples from some of the subjects in this study were independently genotyped in a genome-wide association study to identify susceptibility loci associated with ovarian cancer risk [20] and/or tested for mutations in known ovarian cancer susceptibility genes [21].

### Serum Samples

Blood samples for study biomarker measurements were obtained by routine venipuncture prior to surgery. Samples were allowed to clot and maintained at room temperature during transport. Samples were centrifuged and the serum aliquotted into cryotubes, frozen to −80°C within four hours of sample collection and kept frozen until laboratory analysis.

### Lipid Extraction

Lipids were extracted using a modified Bligh-Dyer method [22], which follows the procedure described below: A mixture was prepared consisting of 1000 pmol DHPE (1,2-Diheptadecanoyl-*sn*-Glycero-3-Phosphoethanolamine), 200 pmol [^13^C_16_] 16∶0 LPA (heavy isotope carbon-13 labeled 1-palmitoyl-2-hydroxy-sn-glycerol-3-phosphatidic acid), and [^13^C_3_] 14∶0 LPC (heavy isotope carbon-13 labeled 1-myristoyl-2-hydroxy-sn-glycerol-3-phosphocholine (N, N, N-^13^C-trimethyl)), which was added to 200 µl patient serum, collected as described above. These added lipids served as internal standards for the quantification of LPA, PPE, and LPC, respectively. The mixture was vortexed and 2 ml 2∶1 (v∶v) methanol-chloroform was added. The new mixture was vortexed again and kept at room temperature for 10 min. and then was centrifuged at 3000 g at 10°C for 10 min. After centrifugation, two layers could be seen in this mixture. The top layer is a mixture of water, methanol, and chloroform while the bottom layer is a proteinaceous pellet. The top liquid layer was transferred into another tube and dried under nitrogen. The dried pellet was reconstituted in 200 µl 0.1 M ammonium acetate dissolved in methanol and transferred into an injection insert inside an injection vial.

### Chromatography and Mass Spectrometry

Liquid chromatography electrospray tandem mass spectrometry (LC/ESI/MS/MS) analyses of LPA, PPE, and LPC were performed using a Quattro Micro mass spectrometer (Waters, Milford, MA, USA) equipped with an electrospray ionization (ESI) probe and interfaced with a Shimadzu SCL-10Avp HPLC system (Shimadzu, Tokyo, Japan).

For LPA and PPE quantification, the lipids were separated with a Luna 5μ C18(2) column (50×2.0 mm, 5 µm of particle size, Phenomenex, Torrance, CA, USA). 1 mM ammonium acetate aqueous solution was used as mobile phase A while 1 mM ammonium acetate dissolved in methanol was used as mobile phase B. The total run time was 15 min and the flow rate was 200 µl/min. The gradient used was as follows: the column was first equilibrated with 20% B (80% A), followed by a linear change from 20% B (80% A) to 100% B (0% A) in the first 3 min. The gradient was kept at 100% B (0% A) in the following 8 min. In the remaining 4 min, the gradient was changed back to 70% B (30% A) to re-equilibrate the column. To reduce the contamination of the mass spectrometer, the eluants between 0–2.5 min and 13–15 min were directed into waste. 20 µl sample was injected into a 50 µl injection loop. Mass spectrometric analyses were performed online using electrospray ionization tandem mass spectrometry in the negative multiple reaction monitoring (MRM) mode. The MS parameters are: capillary voltage, 3.0 KV; cone voltage, 50 V; source temperature, 100°C; desolvation temperature, 350°C; flow rate of desolvation gas, 500 L/hr; flow rate of cone gas, 50 L/hr; mass resolution of both parent and daughter ions, 15.0; multiplier, 650.

For LPC quantification, the lipids were separated with a Hypersil GOLD DASH HTS column (20×2.1 mm, 5 µm of particle size, Thermo Electron, Waltham, MA). 0.3% formic acid in water was used as mobile phase A while 0.3% formic acid in methanol was used as mobile phase B. The total run time was 14 min and the flow rate was 200 µl/min. The gradient used was as follows: the column was first equilibrated with 20% B (80% A), followed by a linear change from 20% B (80% A) to 100% B (0% A) in the first 3 min. The gradient was kept at 100% B (0% A) in the following 7 min. In the remaining 4 min, the gradient was changed back to 20% B (80% A) to re-equilibrate the column. To reduce the contamination of the mass spectrometer, the eluants between 0–2.5 min and 11–14 min were directed into waste. 40 µl sample was injected into a 20 µl injection loop. Mass spectrometric analyses were performed online using electrospray ionization tandem mass spectrometry in the positive multiple reaction monitoring (MRM) mode. The MS parameters are: capillary voltage, 4.0 KV; cone voltage, 40 V; source temperature, 100°C; desolvation temperature, 350°C; flow rate of desolvation gas, 500 L/hr; flow rate of cone gas, 50 L/hr; mass resolution of both parent and daughter ions, 15.0; multiplier, 650.

### Laboratory Quality Control Measures

Because batch effects are a potential issue in biomarker discovery efforts, we have employed several strategies to monitor and account for this concern, including internal standards, quality control samples and calibrators. The internal standards are described above (under “Lipid Extraction”). Quality control (QC) samples consisted of multiple identical 300 µL aliquots of pooled human serum. One QC sample was run before every 10 clinical samples. Calibrators consisted of varying concentrations of pure lipid standards spiked into 4% human serum albumin in DPBS buffer. Nine different concentrations of calibrators were made from the stock solution and run prior to each 100 samples.

### Data Pre-processing and Normalization

Data obtained from the three quality control strategies described above were used to address run-to-run variation and provide a basis for conversion of chromatography peak area to absolute concentration for each analyte. The areas under the chromatography peaks of 8 PPEs, 6 LPAs, 5 LPCs and their corresponding internal standards (labeled by heavy isotopes) were obtained. The areas under the peaks of the internal standards were used to estimate the peak ratio between the analyte and the internal standards. The behavior of each analyte in peak generation compared with its corresponding internal standard was similar, with the exception of SPC and PAF-C16; these analytes were excluded from the analyses. Thus, a total of 17 lipids and CA125 were included in the analyses.

Let *R* denote the peak ratio between the area under the chromatographic peak for analyte over the internal standards, and *C* denote the concentration. Given the observed linear relationship between the quantified peak ratio and known concentration in each calibrator data set, a simple linear regression was performed to estimate the regression slope for each batch, 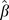, which was then used for concentration conversion for the clinical samples from the peak ratio as 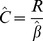. We used the QC sample run between each 10 clinical samples to examine the variation of any potential batch effect. The estimated QC concentration was also used to calculate the batch-adjusted concentrations for each analyte so that the concentrations were comparable across batches for each of the LPAs and PPEs. If the adjusted concentration was lower than zero, then zero was used for the estimated concentration. For LPCs, there was no observable indication of batch effects. Therefore, no batch adjustment was performed.

### Development of Statistical Models

We employed a powerful statistical and machine learning approach, SVM [16], [17], to classify the 211 samples of the patients diagnosed with EOC (cases) and the 212 benign samples (benigns) included in the analysis. Although SVM demonstrates a superior classification performance to many other statistical and machine learning methods, it also shares some common challenges with other methods, namely, variable selection (biomarker selection) and the risk of generating overly optimistic error rates due to model overfitting. We used HH-SVM [18] to first prioritize markers to enter the model for classification between benigns and cases. HH-SVM combines the huberized hinge loss function and the elastic-net penalty to perform classification and variable selection. We used the previously published *R* code [18] to generate weight plots to prioritize the markers based on their importance in classification. Because serum CA125 is a common biomarker used in clinical practice, we built the models by including CA125 and adding the candidate marker lipids one-by-one, based on the weights estimated by HH-SVM. Each of the SVMs was fitted using Matlab function *svmclassify* and the parameters were estimated. We used *K*-fold cross-validation (CV) to avoid model overfitting [19], where *K* = 5 in our study. The generalized errors estimated using 5-fold cross-validation is more objective [19] than the error rates estimated without cross-validation. The data were split into *K* ( = 5) roughly equal-size parts. For the *k* ^th^ (i.e., the 5^th^) part, there is roughly an equal portion of EOC case and benign samples (half and half) and the model was fitted to the other *K*-1 ( = 4) parts of the data. The prediction error of the model was then calculated for the *k*th part. The procedure was carried out for *k* = 1, 2,…, 5, and then the cross validation estimate of the prediction error (CV errors) was computed as
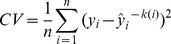
where 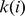 is the part containing observation *i*, and 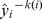 is the fitted value for observation *i*, computed with the 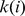 ^th^ part of the data removed.

We developed two types of models, one-step models and two-step models. For the one-step models, we did not use a specific cutpoint for CA125 values although CA125 values were included in the SVM models. For the two-step models, we developed our algorithms for the high and low risk groups by first using the CA125 cutpoint of 35 units/ml, since this is the most commonly reported reference value used clinically to designate a positive test, and has also been used in screening trials to define an abnormal test result, including in the Prostate, Lung, Colorectal, Ovarian Cancer Screening Trial [13].Therefore, we used the simple cutpoint of 35 units/ml as the first step in building the two-step models. Note that all patients in our study underwent diagnostic radiologic imaging, not screening, and imaging information is not incorporated into the model development.

For the one-step models, for a given set of markers, the model is developed in the same manner for all samples, regardless of the preoperative CA125 level. We began the model development with CA125 alone. Candidate phospholipids were ranked by the weights generated using HH-SVM as described above, and then added into the models one by one during the model development. Using cross-validation is more conservative when compared with the models developed without cross-validation. For proof of principle, we developed both models with and without cross-validation, illustrating that the models with cross-validation yield more objective error rates.

For the two-step models, the samples were first classified into high-CA125 (CA125≥35 units/ml) and low-CA125 (CA125<35 units/ml) groups. In the second step, we developed separate models for each of the two groups, the high- and low-CA125 groups, respectively. Specifically, we developed the two-step models based on the commonly reported cutpoint of CA125 at 35 units/ml as the first step; we then queried whether a second test added to this reference value of CA125 can improve diagnostic accuracy for classifying “cases” (samples from women with EOC) and “benigns” (samples from women without cancer).

## Results

The current study included samples from all participants in whom a diagnosis of EOC was confirmed (N = 233) and an equal number of randomly selected samples from women from the same cohort diagnosed with benign disease. Two cases were excluded due to unavailability of data on preoperative CA125 level. Preliminary analysis (using boxplots and histograms; data not shown) suggested that levels of the measured biomarkers may potentially differ between different racial groups, and only a small portion of samples are from non-Caucasian subjects. Therefore, we limited the analysis to Caucasian subjects. This resulted in a total of 211 EOC samples and 212 benign samples included in the analyses. Among the 211 EOC cases, the mean age (±standard deviation) was 62 (±12) years old, with 31 premenopausal and 180 postmenopausal. Among the 212 benigns, the mean age (±standard deviation) was 57 (±14), among whom 65 were premenopausal and 147 postmenopausal. Further details regarding characteristics of the EOC cases are provided in Table 1.

**Table 1:** Histopathology of epithelial ovarian cancer cases.

Clinical data for cases (n = 211)		
Characteristics	Stages I and II (n = 78)	Stages III and IV (n = 133)
Tumor Category		
Carcinoma (n = 179)	48 (22.3%)	131 (62.6%)
Borderline (n = 32)	30 (14.2%)	2 (0.9%)
Stage		
I	60 (28%)	–
II	18 (8.5%)	–
III	–	116 (57.8%)
IV	–	17 (5.7%)
Grade		
1	39 (18.5%)	2 (1.4%)
2	7 (2.8%)	8 (3.3%)
3	30 (14.7%)	122 (58.3%)
Ungraded	2 (0.5%)	1 (0.5%)
Histological Type		
Serous	35 (16.6%)	108 (50.7%)
Endometrioid	14 (6.2%)	4 (2.4%)
Mucinous	14 (6.6%)	2 (0.9%)
Clear cell	4 (1.9%)	5 (2.4%)
Mixed	10 (4.7%)	9 (4.2%)
Unknown	1 (0.5%)	5 (2.4%)

### CA125 results

A total of 211 cases and 212 benigns was included in the analysis. Using only the CA125 concentration with cutpoint of 35 units/ml to classify samples into cases (CA125≥35 units/ml) and benigns (CA125<35 units/ml), the error rate is 29.79% (126/423). The sensitivity is 84.36% (178/211) and the specificity is 56.13% (119/212). The false positive rate (benign samples classified as cases) is fairly high, as is typical in clinical practice.

### One-step Model Results

We began the model development with CA125 alone, the only commonly used clinical biomarker for ovarian cancer. Additional markers were added into the models one-by-one based on the weights estimated using HH-SVM (Table 2).

**Table 2:** Biomarkers ranked according to weights generated by Huberized Hinge Support Vector Machine (HHSVM) for classification of benigns and cases.

Biomarkers	Weight
16∶0, 18∶1 PPE	0.415
18∶2 LPA	0.379
CA125	0.286
15∶0 LPC	0.148
14∶0 LPC	0.137
20∶4 LPA	0.124
16∶0, 18∶2 PPE	0.076
16∶0 LPA	0.067
18∶1 LPA	0.058
22∶6 LPA	0.046
18∶0, 18∶1 PPE	0.045
18∶0, 18∶2 PPE	0.040
18∶0, 22∶6 PPE	0.029
18∶0, 20∶4 PPE	0.009
18∶0 LPA	0.008
16∶0, 22∶6 PPE	0.005
12∶0 LPC	0.004
16∶0, 20∶4 PPE	0.003

The weight was estimated for each marker using HHSVM to indicate its relevance in the classification. Higher weight indicates more importance of the variable (biomarker) in the classification. Abbreviations used:lysophosphatidic acid (LPA), lysophosphatidylcoline (LPC) and plasmenylphosphoethanolamine (PPE).

As described in the methods, one set of models were fitted with CV, and the other without CV. The estimated error rates for models with 1 to 5 included markers are summarized in Table 3. As expected, the models without CV have more optimistic estimates of error rates. As the number of variables increases, the error rates become smaller. In contrast, the CV error of the models with two markers, CA125 and 16∶0, 18∶1 PPE, is the minimum among the 5 models fitted with CV (Table 3). As the number of markers increases from 2 to 5, the CV errors become larger (instead of smaller). The CV errors increase as the number of markers increases and indicate the overfitting as expected. The models with higher numbers of markers are not listed here. The model with minimum CV error contains two markers, CA125 and 16∶0, 18∶1 PPE. Its improvement for classification over the model with only CA125 is due to the improvement in specificity. The specificity is 85.38% and the sensitivity is 70.62%. To compare the performance of the model to that of the cutpoint of 35 units/ml for CA125, setting the specificity to 56.13%, the sensitivity is improved from 84.36% to 88.15%. In a similar fashion, using this model, there is comparable diagnostic specificity of 58.49% (compared to 56.13%) in this set of samples, while maintaining the sensitivity at 84.36%.

**Table 3:** Estimated error rates for the support vector machine models for classifying benigns and cases.

Number of biomarkers	1	2	3	4	5
**Biomarkers**	CA125	CA125, 16∶0 18∶1 PPE	CA125, 16∶0 18∶1 PPE, 18∶2 LPA	CA125, 16∶0 18∶1 PPE, 18∶2 LPA, 15∶0 LPC	CA125, 16∶0 18∶1 PPE, 18∶2 LPA, 15∶0 LPC, 14∶0 LPC
**% Error rates without CV**	21.75	21.51	20.33	17.73	13.95
**% CV errors**	22.22	21.99	24.11	24.35	25.30

. One set of models was fitted with cross-validation (CV) and the other without.

### Two-step Model Results

In the one-step models above, we have shown that the model development using cross-validation is more conservative. Thus, for the two-step models, we used only the conservative approach to develop models in order to avoid overfitting. As described in the section Materials and Methods, the samples were first classified into high-CA125 (CA125≥35) or low-CA125 (CA125<35) groups. For the second step, we developed separate SVM models for the high-CA125 and low-CA125 groups. The HH-SVM as described in the Materials and Methods section was used to prioritize biomarkers for the high- and low-CA125 groups, respectively.

Based on the ranking of the markers in each of the high- and low-CA125 groups (Table 4), the cross-validation errors for SVMs with 1 to 7 markers were estimated, respectively. For the low CA125 group, the first SVM model contains the top-ranked marker, 16∶0, 18∶1 PPE. The model was first fitted and CV error was estimated. The second SVM model contains an additional marker in the model, 15∶0 LPC, in addition to the original (highest-ranked) marker, 16∶0, 18∶1 PPE. This procedure of adding markers one-by-one into models based on the weights estimated by HH-SVM is repeated for the models with 3 markers, 4 markers, and up to 7 markers. As the number of markers in the model increases, the increased CV errors indicate the overfitting of models as expected, so we stopped adding markers. For the low-CA125 group, the model with the smallest CV error rate is the model with 4 markers: 16∶0, 18∶1 PPE, 15∶0 LPC, 18∶2 LPA, and 18∶0, 22∶6 PPE (as part of all 3 models in Table 5), referred to as “4 marker set” in Table 5. For the high CA125 group, the models were fitted in the same fashion. The first SVM contains the top-ranked marker, 16∶0, 18∶1 PPE, and additional models with more markers were built by adding them one-by-one. The model was first fitted and CV error was estimated. The smallest CV error rate is that of the model with 2 markers: 16∶0, 18∶1 PPE and 14∶0 LPC, (as part of model *M3* in Table 5). These 2 markers are referred to as “2 marker set” in Table 5.

**Table 4:** The weights generated by Huberized Hinge Support Vector Machine (HHSVM) to indicate the relevance of the biomarkers for classification of benigns and cases for the samples with either low- or high- CA125 levels (CA125<35 or CA125≥35) in the second step of the development of the two-step models.

	*Low CA125*		*High CA125*	
Analyte	Ranking	weights	Ranking	weights
16∶0 18∶1 PPE	1	0.899	1	0.281
15∶0 LPC	2	0.277	15	0.010
18∶2 LPA	3	0.217	3	0.263
18∶0 22∶6 PPE	4	0.172	5	0.078
14∶0 LPC	5	0.111	2	0.280
18∶0 18∶1 PPE	6	0.074	8	0.036
18∶0 18∶2 PPE	7	0.063	16	0.003
20∶4 LPA	8	0.044	4	0.090
18∶0 LPA	9	0.035	11	0.027
16∶0 LPA	10	0.030	13	0.017
22∶6 LPA	11	0.019	10	0.029
16∶0 20∶4 PPE	12	0.019	17	0.001
18∶1 LPA	13	0.017	9	0.033
18∶0 20∶4 PPE	14	0.012	14	0.014
12∶0 LPC	15	0.009	12	0.026
16∶0 18∶2 PPE	16	0.003	6	0.067
16∶0 22∶6 PPE	17	0.001	7	0.044

These estimated weights were used to prioritize the markers to enter the model (see the text for more details).

**Table 5:** Sensitivity, specificity and error rates of the selected two-step models.

Models	Markers for low-CA125	Markers for high-CA125	Sensitivity (%)	Specificity (%)	Error rates (%)
*M1*	4 marker set	4 marker set	80.57	69.81	24.83
*M2*	4 marker set	*N/A*	91.94	54.72	26.71
*M3*	4 marker set	2 marker set	75.83	71.70	26.24

The “4 marker set” includes 16∶0, 18∶1 PPE, 15∶0 LPC, 18∶2 LPA, and 18∶0, 22∶6 PPE. The “2 marker set” includes 16∶0 18∶1 PPE and 14∶0 LPC.

The classification error rates, specificities, and sensitivities of the three two-step models with combinations of either the 2 marker sets and/or the 4 marker sets are summarized in Table 3. The first step of the modeling for all three models is identical, i.e., using CA125 concentration of 35 units/ml as the cutpoint to classify samples into high- and low-CA125 groups. Different models for the high- and low-CA125 groups were fitted separately (Table 5). The first model, *M1*, has separate SVM models for the high- and low-CA125 groups. The markers for each SVM are the 4 markers mentioned above (16∶0, 18∶1 PPE, 15∶0 LPC, 18∶2 LPA, and 18∶0, 22∶6 PPE), but the models are fitted separately for each group of samples.

The second model, *M2*, consists of only SVM model for the low-CA125 group, and no additional models for the high-CA125 group (other than the simple cutpoint using CA125). When setting the specificity at the level of 56.13% (derived using the cutpoint of 35 units/ml for CA125), the sensitivity is 90.05%, an improvement over the performance of using CA125 alone (84.36%). The third model, *M3*, has two separate SVMs - here, not only the models were different, but the included biomarkers were also different. The biomarkers which generated the minimum CV errors for high- and low-CA125 groups, respectively, were used within each group (Table 5).

The second model demonstrates the best performance, improving sensitivity without sacrificing much specificity. Using this model, 16 of 33 cases that were missed by CA125 were identified (see case details in Table 6 below). These cases are primarily early stage and/or mucinous cases. All but one of the cases identified by the additional measurement of phospholipids occurred in post-menopausal women, and the single endometrioid case involved synchronous endometrial cancer. Among the four misidentified endometrioid samples using CA125 alone, one of them was correctly classified by model 2, M2, from the 2-step models (Table 6). Among the three misidentified mixed-epithelial samples using CA125 alone, none of them were salvaged by M2. Among the nine misidentified mucinous samples using CA125 alone, six of them were correctly identified by M2. Stratified analyses by histological subtypes were not performed due to limited sample size by subtypes; there were only 19 mixed epithelial, 16 mucinous, and 18 endometrioid patients in our study.

**Table 6:** Characteristics of cases missed by CA125 using a cutpoint of 35 units/ml and correctly identified/not identified by model 2 (M2) using phospholipid measurements.

Stage	Menopausal status	Tumor Type
Cases correctly classified by phospholipid measurement		
1a	Post	Endometrioid
1a	Post	Mucinous
1a	Post	Mucinous
1a	Post	Mucinous
1a	Post	Mucinous
1a	Post	Mucinous
1a	Pre	Mucinous
1a	Post	Mucinous
1a	Post	Serous
1a	Post	Serous
1a	Post	Serous
1a	Post	Serous
2a	Post	Serous
3b	Post	Serous
3c	Post	Serous
3c	Post	Serous
Cases not correctly classified by phospholipid measurement		
1a	Post	Clear cell
1a	Post	Endometrioid
1a	Post	Mixed epithelial
1a	Pre	Mixed epithelial
1a	Pre	Mixed epithelial
1a	Post	Mucinous
1a	Post	Mucinous
1a	Post	Serous
1a	Post	Serous
1a	Post	Serous
1a	Pre	Serous
1c	Post	Endometrioid
2b	Post	Endometrioid
2b	Post	Unknown
3b	Post	Serous
3b	Post	Serous
3c	Post	Serous

Note: The second model, M2, consists of only SVM model for the low-CA125 group, and no additional models for the high-CA125 group. The 4 marker set used in model 2 includes 16∶0, 18∶1 PPE, 15∶0 LPC, 18∶2 LPA, and 18∶0, 22∶6 PPE.

## Discussion

Among the approximately 300,000 women undergoing surgery for ovarian masses in the U.S. each year, there are, unfortunately, few available tools to appropriately triage women with a high likelihood of EOC to gynecologic oncologists for surgery, while allowing the larger group of patients at low risk for malignancy to stay in their community with their primary gynecologist. Findings from the current study suggest that bioactive phospholipids may represent such a clinical tool.

Bioactive lipids have long been recognized to be related to carcinogenesis, rendering them a candidate biomarker class with potential for cancer detection [23], [24]. LLPA, a potent mitogen that induces cell proliferation through the activation of G protein-coupled receptors, was first suggested as a diagnostic marker of ovarian cancer over a decade ago. Subsequent investigations, including our own research, have reaffirmed its potential [14], [25]–[30]. Recently, we discovered that two other classes of lipids, PPE and low-abundance LPC (such as 14∶0 and 12∶0 LPC), are depleted in ovarian cancer serum, suggesting them as additional candidates for ovarian cancer diagnosis and detection.

We measured various LPA, PPE and LPC species in blood samples obtained preoperatively from women with suspected ovarian cancer to assess their potential as diagnostic biomarkers. Our results, obtained through careful experimental design, stringent quality control, and rigorous statistical model development, suggest that the additional measurement of specific phospholipids supplement results of CA125 measurement and improve diagnostic accuracy. Specifically, the observed improvement in sensitivity (identification of cases with CA125 levels below 35) was confined almost entirely to early stage cases and/or those of mucinous histology, both groups in which CA125 performance is historically poor. The potential improvement in sensitivity for early stage cases seems particularly promising, given that opportunities for improved outcomes through improved diagnosis and detection are likely to be higher for early stage cases. Measurement of phospholipids improved the identification of early stage cases from 65% (based on CA125) to 82%, and for mucinous cases from 44% to 88%. Although we had insufficient sample size to stratify analyses by histologic type, this finding is intriguing. Given existing knowledge regarding basic biologic and genetic differences between mucinous tumors and other histologic subtypes [31], [32], the performance of the markers in the mucinous subtype provides additional evidence that our approach is complementary to measurement of CA125.

Our study was by design enriched with challenging benign samples, compared with large screening trials. More specifically, half of our samples are benign samples from women in whom clinical suspicion of EOC was sufficient to warrant surgery; therefore the “false positive” rate of using CA125 at or above 35 units/ml in this study is, as expected, much higher than that of a screening trial. When using CA125 with a cutpoint of 35 units/ml, the specificity in our study is 56.13% and the error rate is 29.79% (126/423). The specificity for large screening trials typically ranges between 97.6% and 99.9% either using CA125 with a cutpoint at 35 units/ml, using serial CA125 measurements, or using serial CA125 combined with ultrasound [12], [33], [34]) because the majority of participants are healthy (non-symptomatic) individuals. Recent research suggests that menopausal status and oral contraceptive use affect baseline CA125 values and that clinical cutpoints of 40 units/ml for premenopausal women on oral contraceptives and 50 units/ml for premenopausal women not using oral contraceptives achieves a 2% false positive rate in ovarian cancer screening trials [35]. We therefore examined these higher cutpoints for premenopausal patients. Employing a CA125 cutpoint of 40 for premenopausal patients and 35 for post-menopausal patients, the sensitivity (83.41%) and specificity (57.55%) are comparable with using the cutpoint of 35 for all patients [sensitivity (84.36%) and specificity (56.13%)]. Using the CA125 cutpoint of 50 for premenopausal patients and 35 for post-menopausal patients, the sensitivity dropped slightly to 81.99% and specificity increased to 63.21%. However, the increased cutpoint could decrease sensitivity, an important clinical consideration [36]. Many factors may influence CA125 levels and further investigation through carefully conducted prospective clinical trials incorporating and evaluating additional factors such as ethnicity and smoking history in addition to OC usage and menopausal status are desirable [36].

The biologic mechanism for depletion of LPCs and PPEs is unknown. PPE is a plasmalogen, a class of lipids with the unique structural feature of a vinyl ether bond at the *sn*-1 position of the glycerol backbone. This vinyl ether bond is vulnerable to oxidation and endows plasmalogens with potent antioxidant capacity. A large body of literature supports the role of oxidation and inflammation in the initiation and progression of epithelial ovarian cancer [37], [38]. Thus, one might speculate that depletion of PPE is a consequence of the oxidative environment associated with EOC. LPC is a major component of the cell membrane. In rapidly proliferating tissues, circulating LPC is recruited from blood for incorporation into new cell membranes, leading to depletion of circulating LPC. However, depletion of common LPC species, such as 16∶0 and 18∶0 LPC may be masked because they are typically replaced rapidly through dietary intake of common foods. On the other hand, low-abundance LPCs, such as 14∶0 and 12∶0, are not readily available through standard dietary intake; thus it may be that their depletion in serum is more readily demonstrated.

Before these lipid markers could be applied clinically, validation of our findings using independent datasets will be required [39]. Furthermore, our current study is limited to Caucasians. It is unknown to what extent these biomarkers and/or cutpoints may apply to individuals from other racial and ethnic groups; investigation in additional populations is clearly needed.

## Conclusion

Our results using rigorous statistical modeling suggest that measurement of specific biologically active phospholipids improves diagnostic sensitivity and accuracy among women with suspected ovarian cancer. Further research will be required to validate these promising findings and to further explore the underlying biologic mechanisms. If validated by independent studies in the future, these biomarkers may prove useful as an adjunct to distinguish among patients at the time of clinical presentation, between ovarian cancer cases and benign conditions with shared symptoms and features.
